# High dose concentration administration of ascorbic acid inhibits tumor growth in BALB/C mice implanted with sarcoma 180 cancer cells via the restriction of angiogenesis

**DOI:** 10.1186/1479-5876-7-70

**Published:** 2009-08-11

**Authors:** Chang-Hwan Yeom, Gunsup Lee, Jin-Hee Park, Jaelim Yu, Seyeon Park, Sang-Yeop Yi, Hye Ree Lee, Young Seon Hong, Joosung Yang, Sukchan Lee

**Affiliations:** 1 Department of Palliative Medicine, Seoul St Mary's Hospital, The Catholic University of Korea, Seoul, 137-701, Korea; 2 Department of Genetic Engineering, Sungkyunkwan University, Suwon, 440-746, Korea; 3 Department of Applied Chemistry, Dongduk Women's University, Seoul, 136-714, Korea; 4 Department of Pathology, Kwandong University, College of Medicine, Goyang, 412-270, Korea; 5 Department of Family Medicine, Gangnam Severance Hospital, Yonsei University College of Medicine, Seoul, 135-720, Korea; 6 Department of Medical Oncology, Seoul St. Mary's Hospital, The Catholic University of Korea, Seoul, 137-701, Korea

## Abstract

To test the carcinostatic effects of ascorbic acid, we challenged the mice of seven experimental groups with 1.7 × 10^-4 ^mol high dose concentration ascorbic acid after intraperitoneal administrating them with sarcoma S-180 cells. The survival rate was increased by 20% in the group that received high dose concentration ascorbic acid, compared to the control. The highest survival rate was observed in the group in which 1.7 × 10^-4 ^mol ascorbic acid had been continuously injected before and after the induction of cancer cells, rather than just after the induction of cancer cells. The expression of three angiogenesis-related genes was inhibited by 0.3 times in bFGF, 7 times in VEGF and 4 times in MMP2 of the groups with higher survival rates. Biopsy Results, gene expression studies, and wound healing analysis *in vivo *and *in vitro *suggested that the carcinostatic effect induced by high dose concentration ascorbic acid occurred through inhibition of angiogenesis.

## Background

Despite advances in medical science, both the number of cancer patients and the death rate due to cancer is increasing. Although new approaches and new carcinostatic agents have been developed, their effects on cancer patients are not sufficient [1]. Since Klenner and colleagues applied vitamin C (ascorbic acid) to cure cancer patients in 1949, cell experiments, model animal experiments and clinical trials have been carried out [2,3]. Linus Pauling and Ewan Cameron reported that the administration of high dose concentrations of ascorbic acid (1.7 × 10^-4 ^mol) to cancer patients in the terminal stage improved the quality of life and extended their lives [4]. Although there are experimental results supporting the carcinostatic effects of ascorbic acid and its use as a therapeutic agent to prevent the growth of cancer cells, there is still controversy over the effects of ascorbic acid. According to the work done by Levin's group [5,6], ascorbic acid has definite effect as an antitumor agent when administrated at a high dose concentration. They reported that high dose concentrations of ascorbic acid, provided intravenously, work as a pro-oxidant therapeutic agent in cancer by generating ascorbate radicals and hydrogen peroxide in extracellular fluid in vivo. In addition, clinical case reports (from kidney cancer and bladder tumors) strongly indicate that high dose concentration ascorbic acid therapy in cancer treatment should be reassessed. These studies were confirmed by histopathologic review and examined in accordance with National Cancer Institute (NCI) Best Case Series guidelines [7].

Ascorbic acid mediated direct cytotoxicity effects on cancer cells by hydrogen peroxide have been numerously reviewed [8,9] but in some cases the concentration of ascorbic acid radicals and hydrogen peroxide have not been sufficiently induced tumor cell death [6]. Therefore other action mechanism of ascorbic acid as an anticancer drug has been investigated. The one possibility of ascorbic acid mediated angiostatic effects has been recently reported [10,11]. Mikirova and colleagues showed that high dose concentration of ascorbic acid inhibited cell migration ability and gap filling capacity of endothelial progenitor cells (EPCs). Peyman and colleagues showed that ascorbic acid inhibited corneal neovascularization in a rat model. The rat mode was not for angiogenesis study caused by cancer cells but they showed the neovascularization was clearly affected by the concentration of ascorbic acid.

In our recently published works, intraperitoneal administration of a high dose concentration of ascorbic acid quantitatively up-regulated Raf kinase inhibitory protein (RKIP) and annexin A5 expression in a group of BALB/C mice implanted with S-180 sarcoma cancer cells. The increase in RKIP protein level suggested that these proteins are involved in the ascorbic acid-mediated suppression of tumor formation [12].

Based on our previous experiments [12], here we further investigated the non-cytotoxic antitumor activities of ascorbic acid by inhibiting angiogenesis ability in vitro and in vivo. We supported this finding by quantitative real time RT-PCR as well as wound healing assay to examine the expression of three angiogenesis-related genes and the inhibition of angiogenesis in treatment and control groups. This study supports that high dose concentration ascorbic acid treatment inhibits the angiogenesis of cancer cells by one of the antitumor mechanisms triggered by ascorbic acids.

## Methods

### Animals and tumor cell lines

Murine sarcoma S180 cells provided by Korea Cell Line Bank were maintained in RPMI-1640 medium supplemented with 10% fetal bovine serum (Hyclone, Aurora, Canada), 100 U/ml Penicillin-Streptomycin (Hyclone), and Non-Essential Amino Acids (Sigma), at 37°C in a 5% CO_2 _atmosphere. Female BALB/c mouse (Charles River, Seongnam, Korea) weighing 18-22 g were kept under standard laboratory conditions (tap water, constant room temperature 22°C). Principles of laboratory animal care (NIH publication 85-23, revised 1986) were followed and all experiment was carried out under AAALAC International (Association for Assessment and Accreditation of Laboratory Animal Care International) approval.

### Treatments with cancer cells and ascorbic acid

**Figure 1:**
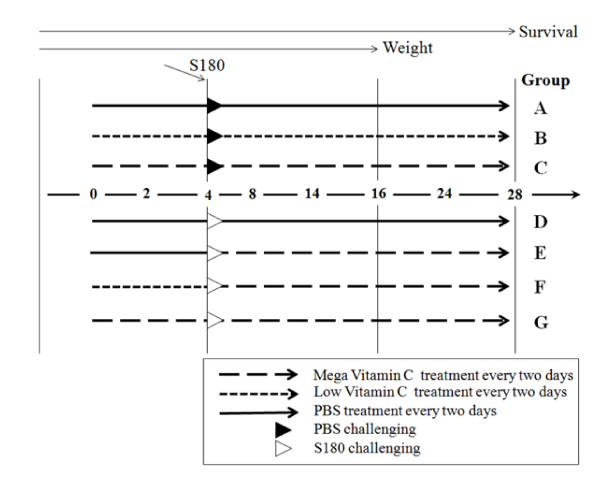
**Schematic diagram for S-180 and ascorbic acid challenge protocol**. Group A: PBS treatment every two days. Group B: Low ascorbic acid treatment every two days. Group C: high dose concentration ascorbic acid treatment every two days. Group D: PBS treatment twice for 4 days and then 5 × 10^5 ^S-180 cells were injected intraperitoneally followed by PBS treatment every two days. Group E: PBS treatment and S-180 cells same as Group D, and then high dose concentration ascorbic acid every two days. Group F: low dose ascorbic acid twice for 4 days, S-180 cells same as Group D, and then high dose concentration ascorbic acid given twice. Group G: high dose concentration ascorbic acid twice for 4 days, S-180 cells same as Group D, and then high dose concentration ascorbic acid given twice. Liver samples of all groups were harvested at 16 days after the first treatment.

Sarcoma 180 cells were cultivated in a CO_2 _incubator for five days, adding 9 ml RPMI 1640 medium, in 8 plates of 100 mm in diameter, and then 5 × 10^5 ^cells in 200 ml PBS were injected into the abdominal cavities of experimental mice using a 21 G injector. The high dose ascorbic acid dose of 1.7 × 10^-4 ^mol (30 mg) corresponds to 100 g for a human of 70 kg. The low dose of ascorbic acid was 3.1 × 10^-5 ^mol (5.5 mg). After each group was treated with ascorbic acid and cancer cells, they were observed and measured over time, and then livers and kidneys were harvested and stored at -70°C for further analysis. BALB/C mice were divided into 7 groups (A - G) with 10 mice per group (Figure 1). Group A was a control group that was treated with phosphate buffer saline (PBS), Group B was treated with low-level ascorbic acid at two-day intervals, and Group C was treated with high dose concentration ascorbic acid at two-day intervals. Group D group was administered Sarcoma 180 cells for cancer induction. Groups E-G received both cancer cells and ascorbic acid. Group E was treated twice with PBS at two-day intervals, injected with S-180 cells, and then treated with high dose concentration ascorbic acid at two-day intervals for Group F was injected with low dose ascorbic acid before injecting cancer cells, and was then treated with high dose concentration ascorbic acid after cancer challenging for 24 days. Group G group was injected with high dose concentration ascorbic acid for four days before injecting cancer cells, and was then treated with high dose concentration ascorbic acid for 24 days after cancer challenging (Figure 1).

### RNA preparation and quantitative real-time RT-PCR

RNA was isolated from livers and kidneys of each group. After evenly grinding the samples from each group, 100 mg of each sample were put in 1.5 ml tubes and 1 ml of Corezol (Corebio System, Seoul, Korea) was added. After adding 200 μl of chloroform to the tubes, we centrifuged them at 12,000 g at 4°C for 15 minutes. The supernatants, which contained the RNA, were placed in new 1.5 ml tubes and then precipitated with 700 μl isopropanol. After centrifuging at 12,000 g at 4°C for 15 minutes, we recovered the RNA pellet in 20 μl DEPCed DDH_2_O [13]. The RNA concentrations were measured by spectrophotometer and electrophoresis. To identify gene expression in the harvested livers, cDNA was synthesized from 5 μg of total RNA using oligo (dT) primers and Moloney murine leukemia virus (MMLV) reverse transcriptase (SuperBio Co. Daejon, Korea). Six ng mRNA was used for reverse transcription. Primers used for quantitative PCR were designed using Primer3  and synthesized by Genotech (Daejon, Korea). Angiogenesis genes detected were bFGF (forward primer: CGG CTG CTG GCT TCT AAG TG; reverse primer: CCC GTT TTG GAT CCG AGT TT), VEGF (forward primer: ACA CGG GAG ACA ATG GGA TG; reverse primer: TCT TGA CTC AGG GCC AGG AA) and MMP2 (forward primer: ATG GGG CTG GAA CAC TCT CA; reverse primer: GGG GCC AGT ACC GTC AG); the housekeeping gene was GAPDH (forward primer: TTG CAG TGG CAA AGT GGA GA; reverse primer: GGC TTC CCG TTG ATG ACA AG). PCR amplification was done in a 20 μl total volume containing 4 or 6 μl of 2 × diluted cDNA (duplicate), 0.25 μM each primer, 1 μl 20000 × diluted SYBR Green I (Molecular Probes, Eugene, OR) and 2.5 units *Taq *DNA polymerase (SuperBio Co. Daejon, Korea) in a reaction buffer composed of 10 mM Tris/HCl (pH 9), 50 mM KCl, 2 mM MgCl_2_, 0.5 mM each deoxyribose trinucleotide, and 0.1% Triton X-100, in a Rotor-Gene 3000 (Corbett Research, Sydney, Australia). PCR cycling parameters were 40 cycles of 10 s at 94°C, 15 s at 60°C, and 20 s at 72°C. The products of real-time quantitative PCR were separated by 1% agarose gel electrophoresis to make sure. Two negative controls, missing either RNA template or reverse transcriptase, were included in each experiment. Each data point represents the average of three experiments and the error bars indicate the standard deviation of individual experiments unless mentioned otherwise.

### Hematoxylin-eosin stain

Specimens were fixed in 10% buffered formalin, serially sectioned, and embedded in paraffin. The prepared paraffin blocks were cut at 3 μm thickness and then stained with hematoxylin-eosin [14].

### Immunohistochemical stain

Representative 3 μm-thick tissue sections for immunohistochemical analysis were mounted on silane coated slides. The sections were deparaffinized in xylene and dehydrated with distilled water through a graded series of ethanol solutions. The slides were pretreated in a microwave oven (20 min) with citrate acid solution for antigen retrieval. After rinsing with APK Wash Solution (Ventana Medical Systems, Tucson, AZ, USA), immunochemistry was performed in a Ventana NexES IHC automated immunostainer (Ventana Medical Systems, Tucson, AZ, USA). The primary antibodies used in this study included MMP-2 and VEGF (ABcam, Cambridge, UK), and bFGF (BD Transduction Laboratories™, San Jose, CA, USA). The prediluted (1:50) primary antibodies were applied for 32 min at 37°C. The sections were then treated for color development with diaminobenzidine (4 min), and counterstaining was done with hematoxylin (4 min) using the iVIEW™ DAB Detection Kit (Ventana Medical Systems).

### Cell migration and cell culture wound assay

We used a wound healing assay [15] to identify the degree of migration of cancer cells and normal cells caused by the treatment with ascorbic acid. Wounds were created in confluent H-ras NIH3T3 cells (Biochemistry laboratory, Department of Genetic Engineering, Sungkyunkwan University) using a pipette tip. The cells were then rinsed with medium to remove any free-floating cells and debris. Serum-free medium was then added, and culture plates were incubated at 37°C. Wound healing was observed at 0, 12, 24, and 36 hours within the scrape line, and representative scrape lines for each cell line were photographed. Duplicate wells of each condition were examined for each experiment, and each experiment was repeated 3 times.

### Statistical analysis

We compared angiogenesis gene expression (bFGF, VEGF, MMP-2), survival rate, and ascites genesis rate between experiment groups. All analyses were carried out using the statistic software Sigmaplot (Systat software Inc. Chicago, USA). Data are presented as mean ± SE.

## Results

### 1. Intraperitoneal cancer progression in each group

**Figure 2:**
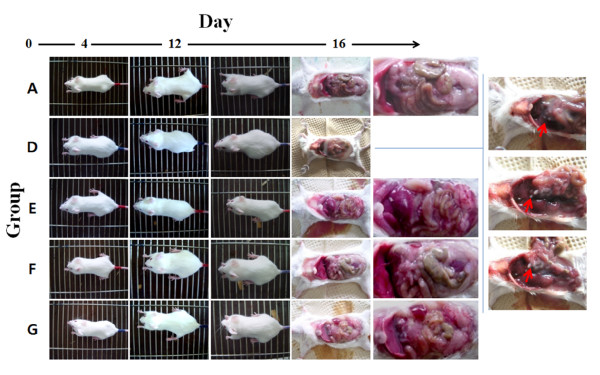
**Effects of high dose concentration of ascorbic acid on mouse model experiments**. Ascite formation and cancer induction were shown in cancer cell injected experimental groups (D to G) with different degrees of ascite formation and cancer induction. Dissection picture of group D shows the most severe ascite formation and polyps, indicated by red arrows.

**Figure 3:**
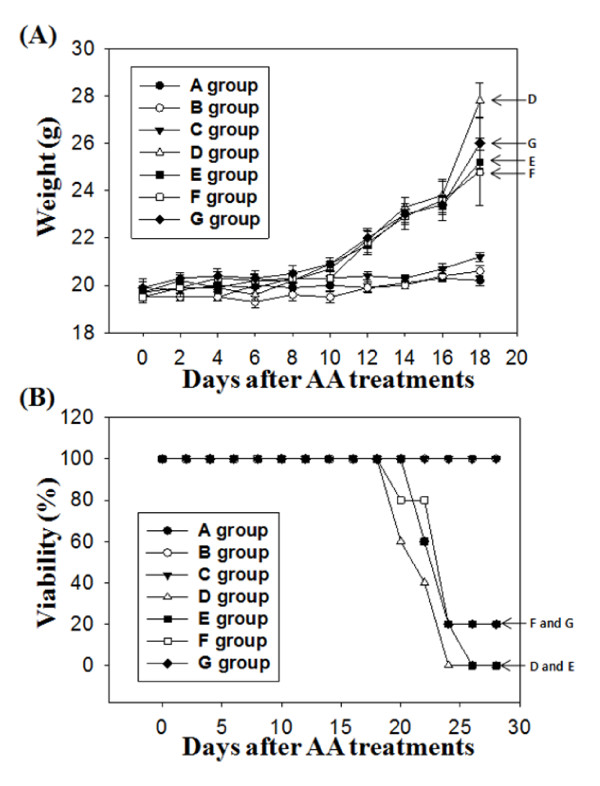
**Ascorbic acid effects in changes of body weight (A) and viability (B) in each experimental group after cancer cell injection**. (A) The body weights were measured from 10 mice of each group up to 18 days after injecting ascorbic acid. (B) Result shows for the changes of survival rates of 5 mice per each group up to 28 days after injecting ascorbic acid.

Sizes of ascites and intraperitoneal tumors were measured at 16 days after ascorbate or PBS treatment (Figure 2). Mice developed ascites containing tumor cells between 6 and 12 days after cancer injection. Group D (no ascorbate treatment) developed intraperitoneal tumors rapidly. Groups E, F and G developed tumors both more slowly and later than Group D. The amounts of ascites were quantified by recording weights of each mouse. The weights of Groups A-C were maintained at about 20 g but Groups D-G increased beginning 6 days after cancer injection (Figure 3A).

Significant tumor induction was observed in Group D compared to the other groups. White masses were formed, indicated by red arrows, in each organ (Figure 2); these were tumors formed by cancer cells. In addition, more ascites were generated in Group D than in the other groups into which cancer cell had been injected.

### 2. Increased viability and decreased acsites production by ascorbic acid treatments

Of the 10 mice in each group, 5 were dissected to measure angiogenesis gene expression and observe abdominal cavities, and 5 mice were observed up to 28 days after injecting ascorbic acid to measure survival rate. Celiectomy was performed around 14 days after the injection of cancer cells, and mice were weighed at that time. The greatest average weight, 27.8 g, was found in Group D at the 14^th ^day after injecting cancer cells, an increase of 1.39 times from the start of the experiment. Groups G, E, and F groups followed in weight order (Figure 3A). The average body weight at the 18^th ^day was 25.2 g of Group E, 24.8 g of Group F and 26 g of Group G respectively. These data showed that the treatments of ascorbic acid by challenging with low dose before cancer infection and then treated with high dose of ascorbic acid was more effective (Group E). At the 18^th ^day, the body weight of Group F was 0.89 times of Group D. Survival rate was measured up to 28 days from the beginning of the experiment. Group D showed a survival rate of 0% after 25 days, and Group E showed a survival rate of 0% after 28 days from the beginning of the treatment. In contrast, F and G groups, which had been treated with ascorbic acid prior to injecting cancer cells, showed a survival rate of 20% at the 28^th ^day (Figure 3B).

### 3. Inhibition of the Expression of Angiogenesis-related Genes by Ascorbic acid

**Figure 4:**
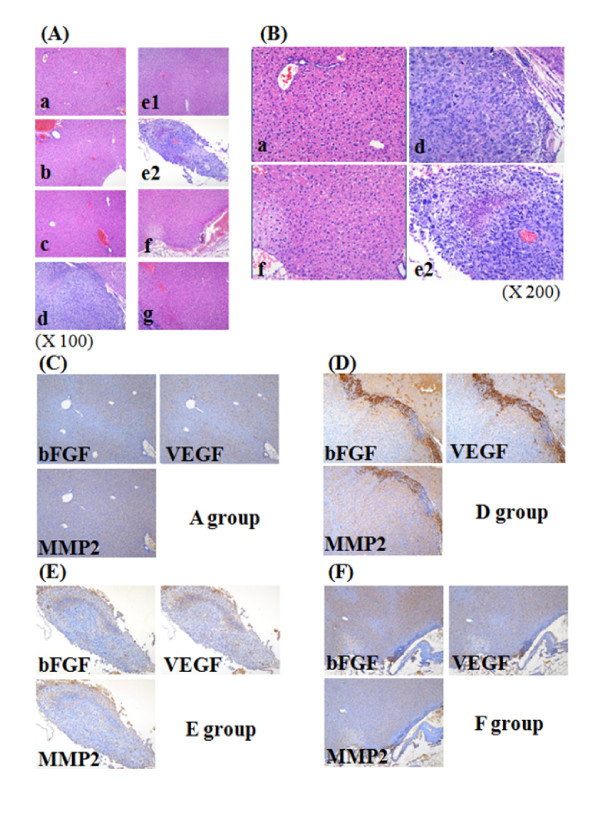
**Tumors in high does ascorbic acid treated groups exhibit poorly formed**. Histochemical (A, × 100 and B, × 200) and immunohistochemical data (C to F) of liver tissues represent the clear tumor staining in group e. A-B: Cancer induction was identified by H & E staining in liver tissues treated with ascorbic acid. Small letters in each figure (a to g) represent the name of each group. C-F: Expression of angiogenesis related proteins (bFGF, VEGF and MMP2) were examined by immunohistochemistry. The name of each tested groups were shown in (C to A group), (D to D group), (E to E group) and (F to F group) in Figures. Angiogenesis related proteins of group D showed dark brown stains rather than other tested groups.

**Figure 5:**
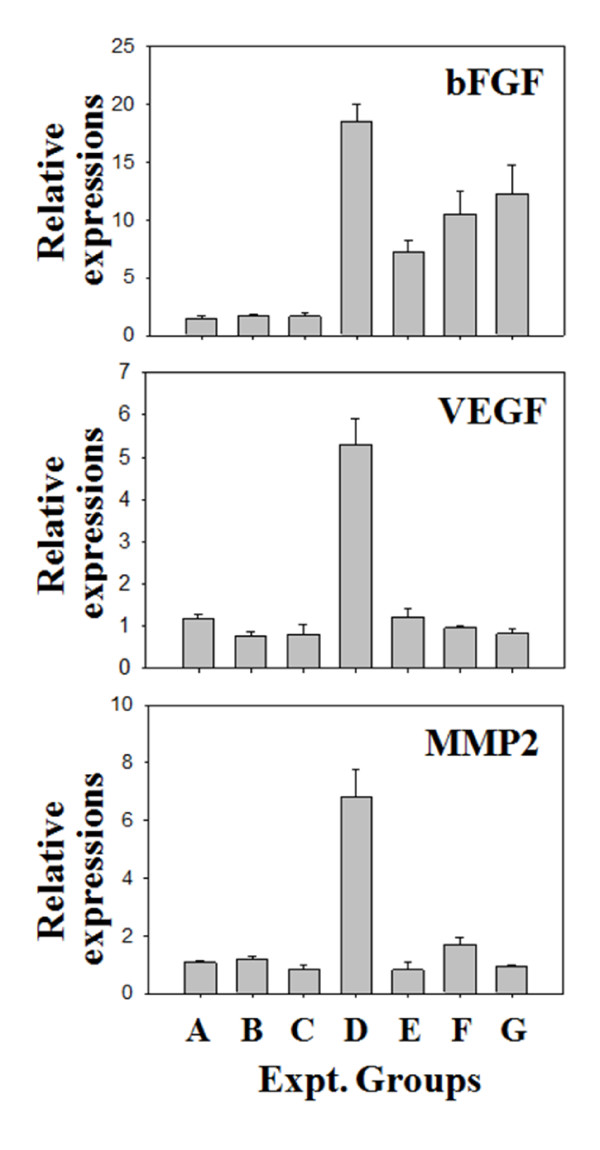
**Quantitative real time RT-PCR (qRT-PCR) analysis of the three angiogenesis related genes**. Expression patterns of three angiogenesis related genes (bFGF, VEGF and MMP2) were high in group D and it is correlated with the immunohistochemistry analysis. Ascorbic acid treated groups showed suppressed expression of these genes. Each qRT-PCR is a representative example of data from 3 replicate experiments.

Angiogenesis is an important mechanism in cancer genesis and the growth process. We measured gene expression of genes involved in angiogenesis by staining and real-time PCR. Cancer genesis in each group was identified by H&E staining as shown in Figure 4A. We observed blue staining of giant nuclei followed by cancer cell genesis, and identified cancer genesis in tissue from Group E (Figure 4B, e2). No staining was found in the other groups; they did not differ from the negative control groups to which cancer cells had not been injected. Thus there was a remarkable reduction of cancer genesis in the groups which received prior treatment with ascorbic acid. We also measured gene expression involved in angiogenesis by immunohistochemistry (Figure 4B). Additional histochemical staining was made for A, D, E, and F groups. As a result of staining with antibody of other 3 angiogenesis related protein, applied to this test, in each tissue, no histochemical staining was made in other groups except D group (Figure 4B). We also analyzed expression of genes involved in angiogenesis by Quantitative real-time RT-PCR (Figure 5). In Group D, expression of bFGF was increased by about 18 times over the groups that did not receive injected cancer cells. This increase was 2.5 times, 1.8 times, and about 1.3 times greater than the increase seen in Groups E, F, and G, respectively, groups which had been treated with ascorbic acid after injecting cancer cells. In Group D, expression of VEGF was increased by 4.5-7; for MMP2, the increase was about 5 times. The expression of angiogenesis related genes was thus remarkably reduced in the groups with ascorbic acid treatment compared to the group with cancer cell treatment only. These results suggest that ascorbic acid treatment in high concentration inhibits angiogenesis by inhibiting the expression of angiogenesis related genes.

### 4. Inhibition of Cancer by Ascorbic Acid in H-ras NIH-3T3 cells

**Figure 6:**
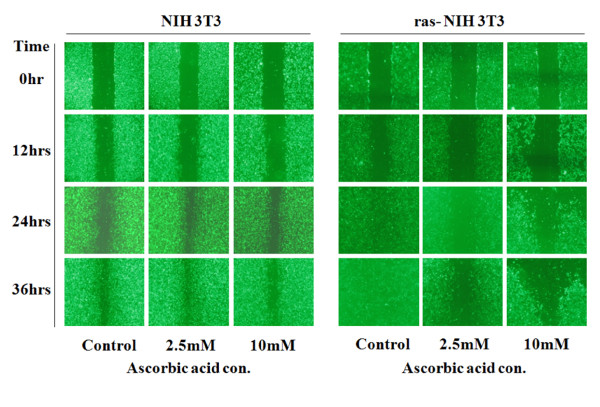
**Wound healing assay on NIH3T3 and ras-NIH3T3 cells depending on the concentration of ascorbic acids and the treated times**. The cell migration of ras-NIH 3T3 cells was inhibited by the treatments of ascorbic acid (2.5 mM and 10 mM), 24 hours after treatments.

We used a wound healing assay to compare the inhibition of the expression of angiogenesis related genes and protein synthesis by ascorbic acid with the change of cell migration efficiency (Figure 6). We observed wound recovery at 0, 12, 24, 36 hrs after treating with 2.5 mM or 10 mM ascorbic acid. The H-ras NIH3T3 cells did not recover after wounding and high treatment concentration of ascorbic acid, while artificially formed wounding was recovered in NIH3T3 cell at 12, 24, 36 hrs by cell migration even in ascorbic acid in 2.5 mM and ascorbic acid in 10 mM (Figure 6). Therefore, migration was inhibited according to ascorbic acid concentration in cancer cell and the treatment time.

## Conclusion

Ascorbic acid is known to be a nontoxic substance. Demol (1934) injected 5 g/kg into guinea pigs, but no specific adverse reaction was found. The above amount corresponds to 350 g for a human of 70 kg. In our research, no specific adverse reaction was observed in control groups (A, B, and C). Several adverse effects have been hypothesized to occur from administration of high dose concentration ascorbic acid; however, these are only known from in vitro experiments or single case reports in most cases. These adverse reactions include genomic mutation, birth defects, cancer, arteriosclerosis, Calculus of kidney, rebound scurvy, oxidative stress, hyperabsorption of limatura ferri, deficiency in ascorbic acid B_12_, and erosion of enamel [16]. However, there is no scientific evidence that high dose concentration ascorbic acid is toxic, harmful, or unfavorable.

Mayland and coworkers (2005) reported that 30% of progressive cancer patients were deficient in blood ascorbic acid [17]. Deficiency in ascorbic acid is related to albumin, platelet, and C-reactive protein (CRP), and it has a negative impact on the prognosis of patients. According to Schorah and colleagues (1996), ascorbic acid concentration in critically ill patients is less than 25% of normal people. In our experiment, injecting ascorbic acid into mice injected with cancer cells led to an increased survival rate over mice injected with cancer cells only, both when ascorbic acid was provided preventively and therapeutically (Figure 3). The group into which ascorbic acid had been injected prior to S-180 cancer cell treatment showed a two times higher survival rate than the group injected with ascorbic acid after S-180 cancer cell treatment (Figure 3).

Angiogenesis related genes are directly involved in the growth and metastasis of tumors. It has previously been shown that expression changes in the angiogenesis related genes bFGF, VEGF, and MMP-2 are closely related to tumor growth and metastasis [18-20]. Therefore we tested that ascorbic acid reduced the expression of three genes (bFGF, VEGF, and MMP-2) when used preventively and/or therapeutically in this experiment. The expression of angiogenesis related genes was lower in the group given ascorbic acid prior to S-180 cancer cell treatment than the group which received ascorbic acid after induction of cancer cells (Figure 4 and 5). bFGF is related to the growth and shift of endotheliocyte and proteolysis [21-23].; in particular, it makes cancer cells grow by activating FGFR-4 (FGFs including FGF receptor-4) [24]. VEGF induces endothelial growth and increases permeability of cells, so it is frequently observed when tumors form new vessels through which nutrition can be supplied [25-28]. VEGF is expressed more strongly in metastatic cancer, and is less well known in primary cancer than the other genes. The prognosis of metastatic cancer when the primary cancer is not known is worse than for other cancers; thus Karavasilis and colleagues (2005) suggested VEGF as a target for therapy [29]. MMP-2 is known to be involved in the destruction of basement membranes, the most important process of angiogenesis. Therefore if MMP-2 is high, cancer cells can easily invade surrounding tissue, since basement membranes and extracellular matrices are destroyed [30]. Therefore this suggests that ascorbic acid can prevent cancer genesis and metastasis by inhibiting induction and angiogenesis. It appears that ascorbic acid inhibited the activation of cancer cells, invasion into surrounding tissue, or metastasis in the group into which S-180 cancer cells were injected.

Roomi and colleagues (2006) reported similar results from in vitro and in vivo experiments [31-33]. They observed changes in angiogenesis related gene expression as an anticancer effect of ascorbic acid, lysine, proline, arginine, and green tea extract on various cancer cells, and suggested that such substances, including ascorbic acid, were affordable as a cancer remedy. By changing the concentration of ascorbic acid and time it was administered, their experiments uncovered a positive effect on the growth and metastasis of cancer cells in the group to which ascorbic acid had been injected before injecting cancer cells into the abdominal cavity. Past research on the anticancer effects of ascorbic acid had only focused on inhibition of the expression of angiogenesis related genes. Data on administration time and concentration for applying ascorbic acid appears to be fundamental to anticancer treatment in the future. Also Mikirova et al (2008) showed similar observations about anti-angiogenesis effects by high dose concentration ascorbic acid treatment on endothelial progenitor cells in vitro and they suggested that nitric oxide (NO) generation can be one of the mechanism by which ascorbic acid mediated angiostatic effects. Our results also supported the finding shown by Mikirova and Roomi groups and we have demonstrated in vivo and in vitro that high dose concentration of ascorbic acid suppressed the gene expression of angiogenesis-related genes and thereby can inhibit angiogenesis.

According to Ashino and colleagues (2003), cytopermeability is increased by endothelial growth factor and decreased by antioxidant, and ascorbic acid affects angiogenesis through antioxidation reactions and collagen synthesis. Ashino and colleagues also reported that this characteristic of ascorbic acid contributes to resistibility to cancer [34,35]. Ascorbic acid, a strong antioxidant, reduces unstable oxygen, nitrogen, and sulfa active oxygen, and may react as a primary protective mechanism against hydrosoluble active oxygen [36-39]. It would prevent fat-soluble active oxygen by reducing vitamin E. In addition, ascorbic acid prevents the formation of carcinogenic nitrosamines by reducing nitrates through the NAD (nicotinamide adenine dinucleotide)-dependent system [36,38,40]. HIF-1α (hypoxia-inducible factor-1 alpha) is involved in tumor growth as a vector to which cells adapt in hypoxia, and is also involved in cancer metastasis by increasing the expression of other angiogenesis related genes (VEGF) [41]. Ascorbic acid inhibits HIF-1α, through increasing resistibility to cancer [42]. According to our data, high dose concentration of ascorbic acid inhibited the angiogenesis but we could not conclude the action mechanism of ascorbic acid against angiogenesis. This non-cytotoxic action of ascorbic acid would be intensively investigated in further experiments.

An ideal antitumor agent would prevent the growth of cancer cells, extend survival period, and improve the quality of life. Although to date administration of ascorbic acid for people has only been supported by a few clinical research results, recently there has been an increase in the number of research reports on the clinical cases of cured cancer patient [7,43]. The study reported here, based on an animal model, S-180 induced mice, showed both preventive and therapeutic effects of ascorbic acid. Ascorbic acid treatment resulted in reduced expression of angiogenesis related genes involved in the growth and metastasis of cancer as well as increased survival rate. Based on these experimental results, more clinical experiments should be tried, as well as additional research on other cancers.

## Competing interests

The authors declare that they have no competing interests.

## Authors' contributions

CY and KL performed the mouse experiments and gene expression analysis. HL, YH and SY carried out the immunohistochemistry, JP, JY, SP and JY collected and analyzed the wounding healing experiments. CY, KL and SL conceived and designed the experiments and analyzed the data. The manuscript was written by CY, KL and SL. All authors read and approved the final manuscript.
